# Analysis of an accusation of a possible breach of ethics in an article published in Parasite

**DOI:** 10.1051/parasite/2024022

**Published:** 2024-05-15

Parasite is a member of COPE and we pay close attention to the strict observance of the rules of experimental ethics in the articles published in our journal. Our attention has been drawn to a possible breach of ethical rules in an article published in 2018 in Parasite [1], referred to in the remainder of this text as the “article in question”.

The source was an email from the authors of an article published in 2023 [2], referred to in the rest of this text as the “incriminating article”. Upon reading the incriminating article, we noticed that the article in question is not cited in the text itself, but is included in a list of 456 articles given in a Supplementary File [3]; furthermore, the name and email of the Editor-in-Chief of Parasite are not given in another Supplementary File [4].

We convened a Committee to rule on this case, referred to in the remainder of this text as the “Parasite Ethics Committee”. The Parasite Ethics Committee was made up of members of the Parasite editorial team, two members from the office of the Société Française de Parasitologie, and the Managing Director of EDP Sciences. The Parasite Ethics Committee sought the opinion of the Comité de Protection des Personnes de Paris X (Chairman: Prof. Casassus) and then interviewed the authors of the article in question.

On reading the offending article, it appears that the alleged breach of ethical rules attributed to the article in question is a discrepancy between the date of approval by the Comité de Protection des Personnes (Human Protection Committee) and the date of the experiments.

The Parasite Ethics Committee notes that: The experiments carried out in the work reported in the article in question were conducted exclusively on lice. The Parasite Ethics Committee notes that as lice are insects, they are not covered by French laws governing the use of live animals for scientific purposes, which apply only to vertebrates, foetal forms of mammals and cephalopods [5]. On this point, no breach of ethical rules can be attributed.

The authors of the article in question did indeed request an opinion from the Comité de Protection des Personnes, which granted it on the basis of an examination of the protocol for experiments on lice reported in the article. The Parasite Ethics Committee notes that it was not necessary to request this opinion since, as pointed out above, no experiments directly involving human subjects were conducted in this study [5–7].Only patient consent for the scientific use of their lice was required, which was obtained.

In the end, all the authors can be blamed for is having sought approval from a Committee for the Protection of Individuals, which they obtained, even though this was neither necessary nor desirable. Whether they did this within the appropriate time frame or not is therefore irrelevant.

The Parasite Ethics Committee therefore considers that the article in question published in Parasite does not present any ethical issues. The article will remain published and requires no modification.

Apart from this specific case, the Parasite Ethics Committee notes that the incriminating article [2] is not without error: an article [9] cited in one of the supplementary files [3] and noted as having been published by EDP Sciences in Parasite in fact comes from another scientific journal, published by another publisher. This error must therefore be corrected in the offending article.
